# Malignant mesothelioma

**DOI:** 10.1054/bjoc.2001.1763

**Published:** 2001-05

**Authors:** P Ruffié, M Lehmann, F Galateau-Sallé, J L Lagrange, J C Pairon

**Affiliations:** Institut Gustave Roussy, Villejuif; Cancer Research Campaign, Paris; CHU – Hôpital Côte de Nacre, Caen; Hôpital H. Mondor, Créteil; Centre Hospitalier Intercommunal, Créteil, France


British Journal of Cancer (2001) 84(Supplement 2), 49–50
© 2001 FNCLCC
doi: 10.1054/ bjoc.2001.1763, available online at http://www.idealibrary.com on IDEAL®

### Malignant mesothelioma

**P Ruffié[1], M Lehmann[2], F Galateau-Sallé[3], JL Lagrange[4] and JC Pairon[5]**

[1]Institut Gustave Roussy, Villejuif; [2]FNCLCC, Paris; [3] CHU – Hôpital Côte de Nacre, Caen; [4]Hôpital H. Mondor, Créteil; [5]Centre Hospitalier Intercommunal, Créteil, France

Mesotheliomas are primary cancers which develop within serosal cavities, most often in the pleural cavity. Malignant mesothelioma of the pleura is a rare cancer in the general population with an estimated annual incidence of 750 cases. However, in certain industrial regions there are more than 60 cases per million inhabitants. In industrialized countries, mesothelioma is a tumour associated with asbestos. An increased incidence (25% in 3 years) has been seen since 1960, especially in older men. Because of the long latent period between exposure and the development of the disease, the peak incidence is expected to occur between the years 2010 and 2030.

These recommendations were reviewed in May 1999. They will be updated in 2001 or 2002 depending on the publication of new data.

### DIAGNOSIS

An occupational exposure to asbestos must be identified using a standardized questionnaire to identify an occupational history (standard). The assistance of a physician with expertise in occupational diseases can be requested (option). Previous exposure to asbestos can be recognized if pleural plaques are found on thoracic CT scan (although these are not always present).

In cases of doubt, where there is no clear history of exposure, the diagnosis can be facilitated by searching and quantifying asbestos bodies in various biological samples by light microscopy (biometrology). Baseline values have been established to determine if a pulmonary asbestos burden is greater than that of the general population (standard):

- greater than one asbestos body per sputum sample
- greater than one asbestos body per millilitre of broncho-alveolar lavage taken during fibroscopic bronchoscopy
- greater than 1000 asbestos bodies per gram of dry pulmonary tissue (in the case of surgical lung biopsy).

The presence of asbestos bodies in smaller numbers does not exclude the possibility of previous exposure to asbestos. Asbestos fibres in broncho-alveolar lavage or lung tissue samples may be quantified using electron microscopy (option).

A thoracic CT scan is the standard examination to assess tumour extent (and to evaluate response to treatment). To make a diagnosis of pleural mesothelioma, multiple biopsies (with at least 10 samples from multiple sites) of large size (greater or equal to 4 mm), including apparently healthy zones, must be taken at thoracoscopy (standard).

Exploration using video-assisted thoracoscopic surgery is an alternative to thoracoscopy (option). Transparietal biopsy of a solid mass and/or surgical biopsy should be reserved for cases where there is a contra-indication to thoracoscopy and/or videoscopic surgery (option). Surgical or video-assisted surgery is the standard for differentiating a benign organizing pleuritis from a malignant sarcomatoid mesothelioma. Frozen sections should not be done routinely but may help to confirm the presence of diagnostic material.

Immuno-histochemical studies must include as a minimum:

- for epitheloid tumors, cytokeratin (CK 5/6), EMA, calretinin Zymed and two or three negative glandular markers (CEA, CD15, BeREP4...)
- for spindle cell tumours: cytokeratin, vimentin, CD34.

In cases of doubt, a histological diagnosis can be confirmed by sending tissue and clinical samples to a panel of specialized pathologists (French MESOPATH group).

In the absence of clinical symptoms or signs, a search for metastases is not useful (standard).

### CLASSIFICATION

The classification system of the International Mesothelioma Interest Group is currently best adapted to a combined medical-surgical treatment approach, but must also be correlated with the TNM classification of the International Union against Cancer (UICC).

### PROGNOSTIC FACTORS

Three favourable prognostic factors most often identified in multivariate analyses are:

- stage I or II disease (TNM or International Mesothelioma Study Group)
- epithelial histology
- patient performance status 0 or 1.

### TREATMENT

There is no standard treatment (Figure 1). The outcome depends more on the prognostic factors for a given patient than the therapeutic modality chosen.

Chest drain tracts and puncture sites must be routinely and prophylactically irradiated (standard).

Radical surgery as sole treatment has not been validated; it should only be undertaken within a therapeutic study.

Similarly, chemotherapy, immunotherapy alone, immunotherapy combined with chemotherapy or combined approaches

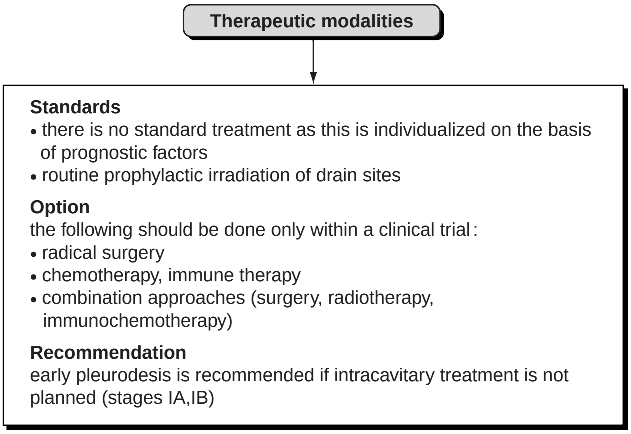

**Figure 1** Treatment of malignant mesothelioma

(surgery, radiotherapy, immunochemotherapy) must only be considered within therapeutic trials.

If intracavitary treatment is not planned (stages IA, IB), early pleurodesis must be undertaken.

### INTERNAL REVIEWERS

V Beckendorf (Centre Alexis Vautrin, Nancy) and P Rebattu (Centre Léon Bérard, Lyon).

### EXTERNAL REVIEWERS

J Ameille (Hôpital Raymond Poincaré, Garches, C Boutin (CHU, Hôpital de la Conception, Marseille), P Chahinian (Mount Sinaï Medical Center, New York, USA), P Delaval (CHR de Pontchaillou, Rennes), MJ Ennem-Simard (Hôpital du Bel Air, Thionville), MC Jaurand (Hôpital Henri Mondor, Créteil), M Letourneux (CHU Hôpital de la Côte de Nacre, Caen), Y Martinet (CHU de Brabois, Vandœuvre-Lès-Nancy), O Menard (CHU de Brabois, Vandœuvre-Lès-Nancy), JC Pairon (Centre hospitalier intercommunal, Créteil), R Riou (Centre Hospitalier, Valence), A Taytard (Hôpital du Haut-Lévêque, Pessac), J Le Treut (Centre Médical Victor Hugo, Pertuis) and P Vuillemin (Centre Saint-Thomas, Aix en Provence).

*© 2001 FNCLCC*

